# Composite versus conventional coronary artery bypass grafting strategy for the anterolateral territory: study protocol for a randomized controlled trial

**DOI:** 10.1186/1745-6215-14-270

**Published:** 2013-08-26

**Authors:** Ariane Drouin, Nicolas Noiseux, Carl Chartrand-Lefebvre, Gilles Soulez, Samer Mansour, Jan-Alexis Tremblay, Fadi Basile, Ignacio Prieto, Louis-Mathieu Stevens

**Affiliations:** 1 Division of Cardiac Surgery, 3840, Saint-Urbain Street, Montreal, Quebec, H2W 1T8, Canada; 2 Department of Radiology, 3840, Saint-Urbain Street, Montreal, Quebec, H2W 1T8, Canada; 3 Division of Cardiology, 3840, Saint-Urbain Street, Montreal, Quebec, H2W 1T8, Canada; 4 Centre hospitalier de l’Université de Montréal (CHUM), CHUM Research Centre (CRCHUM), 3840, Saint-Urbain Street, Montreal, Quebec, H2W 1T8, Canada

**Keywords:** Cardiopulmonary bypass, Composite graft, Coronary artery bypass grafting, Doppler velocimetry, Ischemic heart disease, Left internal mammary artery, Long-term follow-up, Multislice computed tomography angiography, Randomized clinical non-inferiority trial, Saphenous vein graft

## Abstract

**Background:**

In severe coronary artery disease, coronary artery bypass grafting (CABG) surgery is indicated to re-establish an adequate blood supply to the ischemic myocardium. Effectiveness of CABG surgery for symptom relief and mortality decrease should therefore depend on bypass graft patency. As bypass using a left internal mammary artery (LIMA)-to-left anterior descending coronary artery (LAD) anastomosis allows the best results in terms of graft patency, we designed a new surgical technique using a saphenous vein graft as a venous bridge to distribute the LIMA flow to the cardiac anterolateral territory. This novel strategy could extend the patency benefits associated to the LIMA. Other potential benefits of this technique include easier surgical technique, possibility to use saphenous vein grafts as vein patch angioplasty, shorter saphenous vein grafts requirement and reduced or eliminated manipulations of the ascendant aorta (and associated stroke risk).

**Methods/Design:**

Between July 2012 and 2016, 200 patients undergoing a primary isolated CABG surgery using cardiopulmonary bypass with a LAD bypass graft and at least another target on the anterolateral territory will be randomized (1:1) according to 1) the new composite strategy and 2) the conventional strategy with a LIMA-to-LAD anastomosis and revascularization of the other anterolateral target(s) with a separated aorto-coronary saphenous vein graft. The primary objective of the trial is to assess whether the composite strategy allows non-inferior anterolateral graft patency index (proportion of non-occluded CABGs out of the total number of CABGs) compared to the conventional technique. The primary outcome is the anterolateral graft patency index, evaluated at one year by 256-slice computed tomography angiography. Ten years of clinical follow-up is planned to assess clinical outcomes including death, myocardial infarction and need for revascularization.

**Discussion:**

This non-inferiority trial has the potential to advance the adult cardiac surgery field, given the potential benefits associated with the composite grafting strategy.

**Trial registration:**

ClinicalTrials.gov: NCT01585285.

## Background

In severe coronary artery disease, coronary artery bypass grafting (CABG) surgery is indicated to re-establish an adequate blood supply to the ischemic myocardium [1,2]. It reduces morbidity and mortality in patients with left main, triple-vessel disease and/or proximal stenosis of the left anterior descending coronary artery (LAD) compared to medical therapy [2-4], and decreases coronary repeat revascularization rate in comparison to percutaneous coronary intervention [2,5]. Advances in medical therapy for ischemic heart disease and heart failure have improved the outcomes of patients with coronary artery disease. The survival advantage of CABG surgery over medical therapy in patients with stable angina has been challenged [6] and is the subject of the ongoing ISCHEMIA trial (ClinicalTrials.gov: NCT01471522).

Effectiveness of CABG surgery is directly related to graft patency [2,7]. Best graft patency is obtained using the left internal mammary artery (LIMA) for the LAD territory, while saphenous vein graft (SVG) patency is comparatively inferior [2]. In order to expand the LIMA reach to a larger cardiac territory, we conceived a composite CABG strategy: a short SVG used as a venous bridge is sequentially interposed between the LAD and the other target(s) of the cardiac anterolateral territory, and the LIMA is anastomosed on the venous bridge, immediately above the SVG-to-LAD anastomosis (LIMA-saphenous vein bridge strategy; LSVB). In this set-up, the saphenous vein bridge acts as a venous angioplasty at the level of the LAD allowing easier anastomosis of the LIMA for the LAD and secondary distribution of the inflow to the other anterolateral target(s) anastomosed to the saphenous vein bridge. Therefore, this novel CABG technique could allow extension of the LIMA patency advantages while preserving the LIMA flow to the LAD, which was proven important to improve patient survival [8,9].

In a pilot study with 256 patients (mean follow-up of 3 years), we demonstrated the feasibility and security of the new CABG technique [10]. Incidences of clinical events (mortality, myocardial infarction (MI), low cardiac output syndrome, re-exploration for bleeding and recurrence of angina) over the short and long term were low. The graft patency of 20 patients operated according to the LSVB strategy was assessed in a sub-study using 256-slice computed tomography (MSCT) angiography after a mean follow-up of 51 months [10]. The 256-slice MSCT angiography is a non-invasive imaging technique that allows comprehensive and objective assessment of grafts and coronary arteries with an elevated diagnostic accuracy [11-14]. We demonstrated that the LIMA-LAD axis patency index (that is, proportion of non-occluded LIMA-LAD out of the total number of LIMA-LAD) was 100%. The anterolateral patency index (including CABG to the LAD and the other anterolateral target(s)) was 93% compared to the aorto-coronary SVG patency index to other cardiac territories of 87% (*P* = 0.47).

Numerous potential benefits for the composite strategy have been identified during the preliminary studies: 1) SVG-to-LAD anastomoses are less technically complex to perform, compared to LIMA-to-LAD anastomoses when the LAD is severely atherosclerotic, calcified, small or profoundly intramyocardial, or in comparison with sequential anastomoses of the LIMA on a diagonal coronary artery in a side-to-side fashion before joining the LAD with an end-to-side anastomosis, with the risk of compromising the LIMA integrity and distal flow; 2) the SVG can be used as a vein patch angioplasty when the anterolateral coronaries are severely diseased, which is more difficult to perform with LIMA only; 3) manipulations of the ascendant aorta (and associated stroke risk) are reduced, or even eliminated, when the CABG surgery is done as part of an off-pump surgery in which no aorto-coronary graft is needed; 4) arterial conduits can be saved to graft larger territories (that is, territories of the circumflex artery and right coronary artery) than the often smaller territories of anterolateral targets other than the LAD; 5) as the SVG required to revascularize the anterolateral territory is shorter, it is possible to select the most favorable saphenous vein portion to be used as the graft, hence allowing a lower chance of having a diseased SVG portion where the endothelium could have been injured during harvesting; and 6) a more complete revascularization can be performed in patients with saphenous vein or arterial conduits limited supply.

The short- and long-term graft patency results obtained with the conventional CABG strategy are already particularly good [2]; LIMA to LAD and SVG patency index at one year in trials including patients undergoing isolated CABG surgery with cardiopulmonary bypass (CPB) are 96% and 85%, respectively [15-22]. These published graft patencies are comparable to those obtained using other grafting strategies, including total arterial grafting [23,24]. We hypothesized that the LSVB technique could allow a non-inferior, but not necessarily superior, graft patency. As this technique presents the interesting potential benefits stated above, a non-inferiority design was chosen for the study.

To date, few studies discussed the results obtained after a coronary revascularization surgery in which a SVG is used as a composite graft with the LIMA [25-32], and only one exposed a surgical technique somewhat similar to the one we present [28]. Certain surgeons criticize the LSVB technique saying that the interposition of a short portion of SVG between the LIMA and the LAD could be deleterious for the LIMA flow distribution to the LAD, supplying an important part of the left ventricle [33], if the SVG should occlude or should a flow competition establish between the LAD and the other anterolateral target(s). The AMI-PONT prospective randomized clinical non-inferiority trial therefore aims to assess whether the LSVB strategy allows non-inferior results in terms of anterolateral graft patency compared to the conventional CABG technique.

## Methods/Design

### Design

The AMI-PONT trial (ClinicalTrials.gov: NCT01585285) is a prospective single center non-inferiority randomized clinical trial with parallel groups and a 1:1 allocation ratio. The unit of randomization will be the patient while the unit of analysis will be the CABGs, which will be ≥2 by patient (see below).

### Participants

Patients will be eligible if they are more than twenty-one years old and have to undergo a primary isolated CABG surgery with median sternotomy and bypass of the LAD and at least one other suitable anterolateral target. This target can be another site on the LAD, a ramus intermedius, any suitable diagonal branch (diameter >1 millimeter) of the LAD or a high first marginal branch of the left circumflex coronary artery. Targets must present a proximal stenosis of ≥70% (or ≥50% for left main) with an adequate outflow bed according to the surgeon. Patients will be excluded if the LIMA is unusable, such as in uncorrected subclavian artery stenosis, anterior chest trauma or radiation, or injury during harvesting contraindicating its use. Patients with a disease potentially limiting their life expectancy to less than two years, a congestive heart failure with a left ventricular ejection fraction <30%, a contraindication to CPB (that is, calcified aorta) or deemed not suitable for both techniques at the time of surgery by the operating surgeon including planned total arterial revascularisation will not be randomized. Candidates with a contraindication to MSCT angiography will be excluded: moderate to severe renal failure (estimated glomerular filtration rate <50 mL/min/1.73 m^2^), chronic atrial fibrillation, history of severe hypersensibility to an iodinated contrast agent, known or suspected pheochromocytoma, pregnant or breastfeeding female, or severe congestive heart failure (New York Heart Association (NYHA) class IV) unlikely to improve following CABG surgery. At the time of the MSCT angiography, patients will be excluded for the graft patency assessment of the trial if they present persistent rapid (>100 beats/minute) atrial fibrillation or any other cardiac rhythm precluding reliable electrocardiogram (ECG) gating, or a severe congestive heart failure (NYHA class IV) despite coronary revascularization and maximal medical treatment.

**Figure 1:**
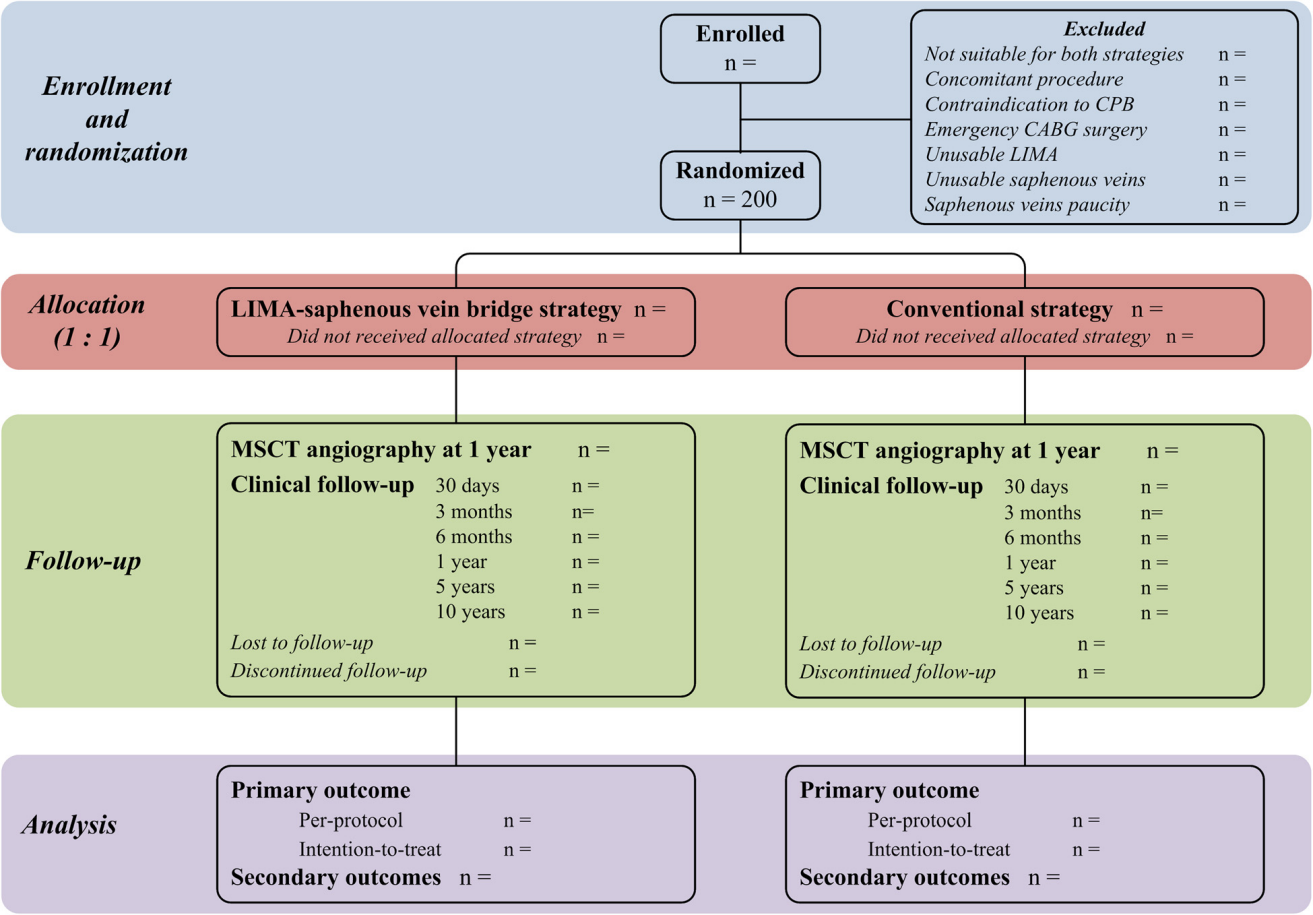
**Study design.** CABG, coronary artery bypass grafting; CPB, cardiopulmonary bypass; LIMA, left internal mammary artery; MSCT, multislice computed tomography.

The AMI-PONT research project was approved by the Centre hospitalier de l’Université de Montréal Research Ethics Committee in April 2012. Eligibility of every patient planned to undergo a CABG surgery at our institution will be assessed. The project will be proposed to every patients meeting the inclusion and exclusion criteria and a written informed consent will be obtained for each participant. The process of enrollment and randomization is showed in Figure 1.

### Randomization: allocation concealment and sequence generation

Randomization will be performed during surgery when the surgeon will confirm, after the sternotomy and exposure of the great vessels, the SVG and LIMA harvesting, and the determination of the anterolateral targets, that it would be safe to perform a CPB surgery using either a conventional or LSVB strategy (the target(s) on the LAD and the other anterolateral target(s) must be aligned in order to avoid a bridge torsion if the patient had to be randomized to a LSVB technique). Randomization will be determined with an opaque sealed envelope. Permuted random blocks of four, six and eight for each participating surgeon will be used and will be stratified in time and by surgeon. The allocation sequence will be created when the research coordinator will draw an envelope in a block associated to the surgeon before the operation of each enrolled patient. Envelopes will be prepared before the enrollment of the first patient.

### Interventions

CABG surgeries will be performed through a median sternotomy using standard CPB with blood cardioplegia under mild hypothermia (34°C) after operative risk evaluation using the Society of Thoracic Surgeons score and EuroSCORE II. Aspirin will be administrated until day of surgery. Clopidogrel and ticagrelor will be discontinued at least 5 days before surgery with prasugrel being discontinued at least 7 days before surgery, unless the surgery is urgent. In order to control for potential bias in patency results according to the use of an on-pump versus an off-pump grafting strategy, all patients will undergo CABG surgery using CPB (on-pump). We recognize that one of the potential benefits of a LSVB grafting strategy is avoidance of ascending aorta manipulation if the surgery is performed without cardiopulmonary bypass (off-pump) and no aorto-coronary grafts are needed, but recruitment of off-pump patients would expose the trial to a potential 5 to 10% conversion rate to on-pump CABG surgery.

**Figure 2:**
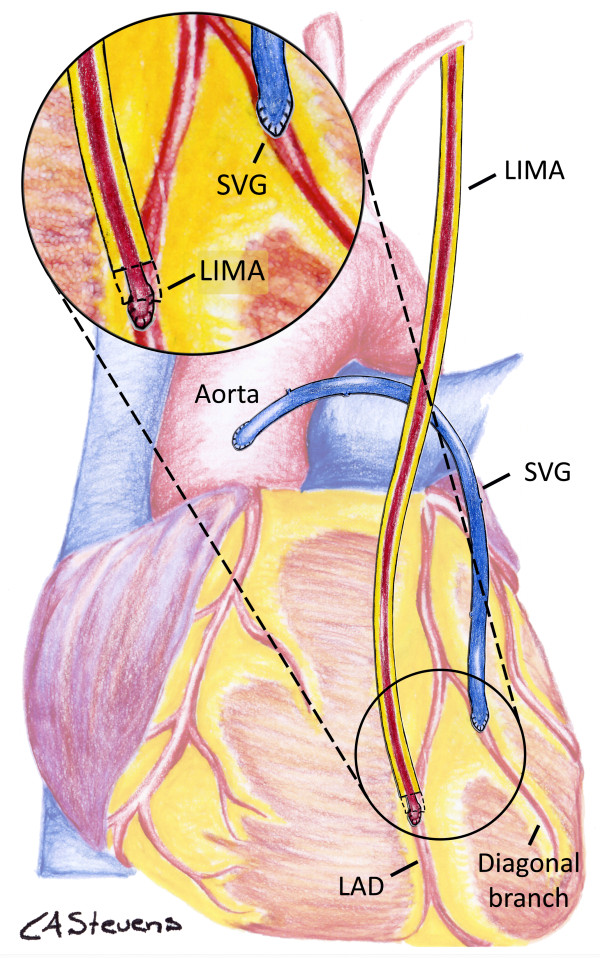
**Conventional strategy.** The *in situ* left internal mammary artery is directly anastomosed to the left anterior descending coronary artery (LAD) and a separated aorto-coronary saphenous vein graft is anastomosed to the anterolateral target(s) other than the LAD. A magnified detail of the distal anastomoses is provided in the left upper corner of the figure. The distal portion of the left internal mammary artery (LIMA) pedicle has been graphically removed in order to present a better view of the distal anastomosis. SVG, saphenous vein graft.

In the conventional strategy (Figure 2), the *in situ* LIMA, harvested in pedicle, will be anastomosed in an end-to-side fashion to the LAD, after the occluded or stenosed coronary portion. If there is only one other target on the anterolateral territory, this target will be bypassed using a SVG anastomosed proximally on the ascendant aorta (that is, aorto-coronary bypass). If there are two or more anterolateral targets other than the LAD, sequential bypass grafts will be completed (that is, more than a bypass by graft, in series) with the sequential SVG anastomosed on the ascendant aorta.

**Figure 3:**
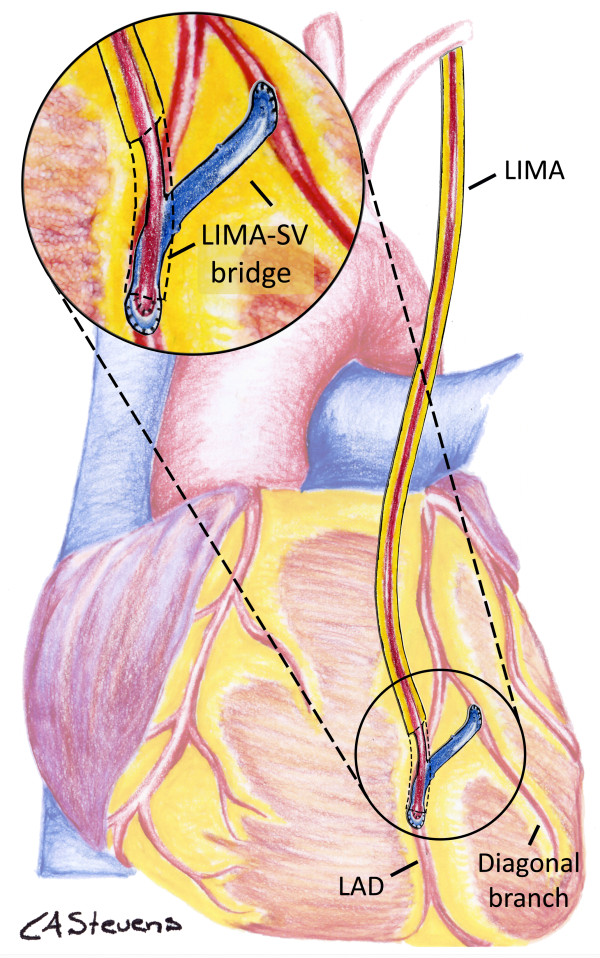
**Left internal mammary artery-saphenous vein bridge strategy.** The *in situ* left internal mammary artery (LIMA) and a saphenous vein bridge are used compositely to distribute the LIMA flow to the anterolateral territory. A magnified detail of the distal anastomoses is provided in the left upper corner of the figure. The distal portion of the LIMA pedicle has been graphically removed in order to present a better view of the distal anastomosis. LAD, left anterior descending coronary artery; SV, saphenous vein.

In the LSVB strategy (Figure 3), a SVG will be used to connect the anterolateral territory targets, including the LAD. If there is only one other anterolateral target than the LAD, a SVG will be anastomosed to these two coronary arteries in an end-to-side manner. If there are two or more anterolateral targets other than the LAD, targets between the LAD and the opposite end of the venous bridge will be anastomosed sequentially to the venous bridge with side-to-side anastomoses. Then, the *in situ* LIMA, harvested in pedicle, will be anastomosed on the venous bridge, as close as possible to the SVG-to-LAD anastomosis, in order to maximally reduce the SVG interposition between the LIMA and the LAD. Hence, blood flow supplied by the LIMA will almost directly supply the LAD, via the interposition of a small portion of SVG, and the other anterolateral target(s) backwards, via the venous bridge.

For both strategies, if other coronary arteries elsewhere than in the anterolateral territory have to be bypassed, CABGs will be performed according to the surgeon preferences, but they will not communicate with the anterolateral grafts. For each coronary target, graft anatomy details will be noted (length, diameter and quality). At the end of the surgery (that is, after protamine administration), graft flow characteristics will be recorded by protocol using a VeriQ Flowmeter System (MediStim ASA, Horten, Norway) for all grafts.

### Objectives

The primary objective of the trial is to assess whether a CABG surgery performed with a LSVB technique allows non-inferior anterolateral graft patency index compared to a conventional strategy.

### Outcomes

The primary outcome is the anterolateral graft patency index, evaluated at 1 year by 256-slice MSCT angiography. Secondary outcomes include 1) patency of each graft, evaluated at 1 year by 256-slice MSCT angiography; 2) freedom from death, new target vessel revascularization (redo CABG surgery or percutaneous coronary intervention) and myocardial infarction at 30 days, 3 months, 6 months, 1 year, 5 years and 10 years; 3) correlation between LIMA, saphenous bridge and SVG transit time Doppler flow data at the time of surgery (VeriQ Flowmeter System, MediStim ASA, Horten, Norway) and graft patency at one year, and clinical outcomes at 30 days, 1 year, 5 years and 10 years; 4) graft lesions severity using a four-point scale. Ten years of follow-up is planned to assess long-term survival.

Clinical events will be ascertained throughout follow-up using predetermined definitions and will be assessed by blinded adjudicators. Clinical events of interest include total mortality, cardiovascular death, recurrence of angina, perioperative (<48 hours) and other postoperative MI, and need for repeated coronary revascularization.

An external Data and Safety Monitoring Board will regularly assess data to insure patient safety according to early- and mid-term clinical outcomes.

### Outcomes measurement

#### Follow-up

Randomized patients will be met in an outpatient visit 30 days after surgery to evaluate occurrence of clinical events, and Canadian Cardiovascular Society (CCS) and NYHA scores. They will then be called 3 months, 6 months, 1 year and 5 years after surgery to assess clinical events occurrence, and CCS and NYHA scores. In addition, clinical data, including cardiac events, re-hospitalizations, procedures and survival status, will be obtained up to twelve years after surgery in order to assure a mean follow-up of ten years, using the provincial health insurance plan registry of the *Régie de l’assurance maladie du Québec* for which patients will have consented at the study onset.

#### Multislice computed tomography angiography

MSCT angiography will be performed at 1 year ± 4 months following CABG surgery. Images will be acquired with contrast-enhanced 256-slice MSCT angiography using prospective ECG-gating and a gantry rotation time of 270 milliseconds. Heart rate control is primordial to obtain superior image quality and for radiation dose reduction. In order to achieve a ≤60 beats/minute heart rate, patients with a heart rate >60 beats/minute will receive an oral β-blocker or calcium-channel blocker. Sublingual nitroglycerine will be given 2 minutes before the MSCT angiography to increase coronary lumen and hence allow greater visualization. Acquired images will be processed using the TeraRecon thin client post-processing software (Aquarius intuition edition version 4.4.4.23.771, Foster City, California, United States).

For interpretation, each graft will be separated into three sections that will be distinctively assessed: graft body, proximal or distal anastomosis, and distal native coronary bed. The image quality of each section will be evaluated before interpretation. A graft will be considered occluded if not opacified on native images and post-processing in the presence of a good opacification of other arterial structures. When assessable, lesion severity will be assessed using a four-point scale: 1) normal lumen (0 to 49% stenosis); 2) moderate stenosis (50 to 69%); 3) severe stenosis (70 to 99% stenosis or string sign (graft diameter <2 mm)); 4) graft occlusion (100%).

### Blinding

Because the surgical technique is different for the two proposed CABG strategies, the surgeon, staff of the operating room and of the postoperative yards, and people collecting the intraoperative data will not be blinded to the performed strategy. Randomized patients will not necessarily be blinded either because in view of the *Public Hospital’s Act and Regulations*, details of the operation must be collected in the patient’s chart when the patient leaves the operating room. Cardiologists assessing clinical events (MI) and the statisticians who will analyze the results will be blinded to the performed surgical technique. The radiologists reading the MSCT angiographies will not be blinded to the surgical mapping, which is readily obvious to them even if they do not have access to it, but they will be blinded to the clinical condition of the patient.

### Sample size

Based on published data, the 1 year LIMA and SVG graft patency index are 96% and 85%, respectively [15-22]. Based on our experience [10], 2.15 grafts on average are required on the anterolateral territory (that is, for about 15% of patients >2 grafts will be performed on the anterolateral territory). We can estimate the graft patency index for all anterolateral grafts in the conventional group with separate LIMA-LAD and SVG to anterolateral coronary targets at ((96% + (1.15 x 85%))/2.15) = 90%. The anterolateral graft patency index in the LSVB group was 93% at a mean follow-up of 51 months [10]; therefore, the expected patency index at 1 year should be ≥93% although most stenosis and occlusions are expected to occur within the first year after surgery. The power analysis is based on a non-inferiority principle, justified by the potential benefits of the LSVB technique, namely increased flow through the LIMA, easier surgical technique and shorter saphenous vein grafts requirement. A predetermined 5% non-inferiority margin was defined based on the smallest decrease in graft patency for anterolateral grafts that the surgeons would be willing to accept considering the identified benefits. Non-inferiority of the LSVB technique will be accepted if the upper bound of the 97.5% confidence interval for the estimated difference in the anterolateral graft patency index lies below 5%. If there is a true difference in favor of the LSVB CABG strategy of 3%, then a total of approximately 430 anterolateral coronary grafts, or 200 patients (randomized 1:1), are necessary to establish non-inferiority of LSVB compared to conventional CABG strategy [34,35] for both the intention-to-treat and per-protocol analyses, assuming a one-sided 2.5% alpha with ≥80% power and a sample size inflation of 10% to account for grafts not analyzable by MSCT, patient dropout and correlated graft patency results in the same patient. This will allow us to be ≥80% sure that the upper limit of a one-sided 97.5% confidence interval (or equivalently a 95% two-sided confidence interval) will exclude a difference in favor of the conventional surgical technique of ≥5%.

### Statistical methods

A two-tailed *P* value of 0.05 will be considered significant. Methodologically, the study will be conducted and reported according to recommended guidelines (CONSORT [36]). For the graft patency outcome assessment, the unit of analysis will be the graft while the unit of randomization is the patient. Sequential grafts will count for as many grafts as there are distal anastomoses. Logistic or ordinal multilevel models, with a random effect for the patient, will be used to account for the correlation between patency results for grafts performed in the same patient. For the primary non-inferiority outcome, patients will be analyzed A) according to the intention-to-treat principle, in which all participants are included in their assigned treatment groups regardless of actual surgical procedure performed, and B) according to per-protocol principle, in which only patients where surgery was completed according to the protocol are included, in order to control for the bias that arises when patient crossover renders patient groups more similar with a greater chance of claiming non-inferiority. Graft patency results will be presented as number and percentage with 95% confidence interval including graphically the predetermined 5% non-inferiority margin. Superiority of the LSVB strategy will also be assessed as a secondary objective if non-inferiority is demonstrated. No multiple-comparison adjustment is required for the two hypotheses tests approach [37]. The intention-to-treat principle will guide the superiority hypothesis test for the main outcome of interest and other secondary outcome analyses [36-38]. Graft occlusion risk factors will be assessed using multilevel logistic regression models. The analyses will also be performed at the level of the patient, assessing the proportion of patients with any occluded bypass graft in the anterolateral territory or elsewhere.

In the clinical outcome analyses, time to event analysis using Kaplan-Meier method, log-rank tests and Cox regression will be the preferred methods. If proportional hazards assumption is violated, we will use both logistic regression and Cox regression with time-dependent covariates with Aalen’s additive hazards model. Participants who prematurely discontinue follow-up before a major cardiovascular event will be censored as to their last follow-up data. The effect of the two operative techniques on different sub-groups (that is, the effect of the two operative techniques on graft patency for different subgroups defined by diabetes, left ventricular function, number of vessels diseased, gender, age, EuroSCORE II (3 to 5 and ≥5) and use of certain medications (clopidogrel and statins) or sequential grafting) will be conducted by stratified analysis through a multilevel logistic regression or multilevel Cox proportional hazards model, as appropriate.

Potential selection bias can be expected from patients excluded from MSCT assessment because of patient refusal, death or loss to follow-up. Sensitivity analysis will be conducted by including excluded patients in the analysis of the percentage of patients with at least one occluded graft, assuming, hypothetically, that the excluded patients present at least one occluded anterolateral grafts. We will assess whether the conclusion reached by this sensitivity analysis is similar to the conclusion reached with the analyses of MSCT patients alone.

## Discussion

### Hypotheses and possible trial outcomes

In non-inferiority trials, the null and alternative hypotheses are reversed compared to superiority trials [36]. Hence, the null hypothesis supposes that there is a difference between the compared treatments and the alternative hypothesis presumes that treatments do not differ, which means that the new treatment is non-inferior to the conventional one(s).

In the AMI-PONT trial, the null (H_0_) and the alternative (H_A_) hypotheses are:

•H_0_: N - C ≤ −Δ (that is, the anterolateral graft patency index in the new grafting strategy group (N) is not non-inferior to the one in the conventional CABG strategy group (C) by a pre-defined non-inferiority margin of -Δ% or less, where Δ = 5 for this trial, or non-inferiority is not shown.)

•H_1_: N - C > −Δ (that is, the anterolateral graft patency index in the new grafting strategy group is non-inferior to the one in the conventional CABG strategy group.)

**Figure 4:**
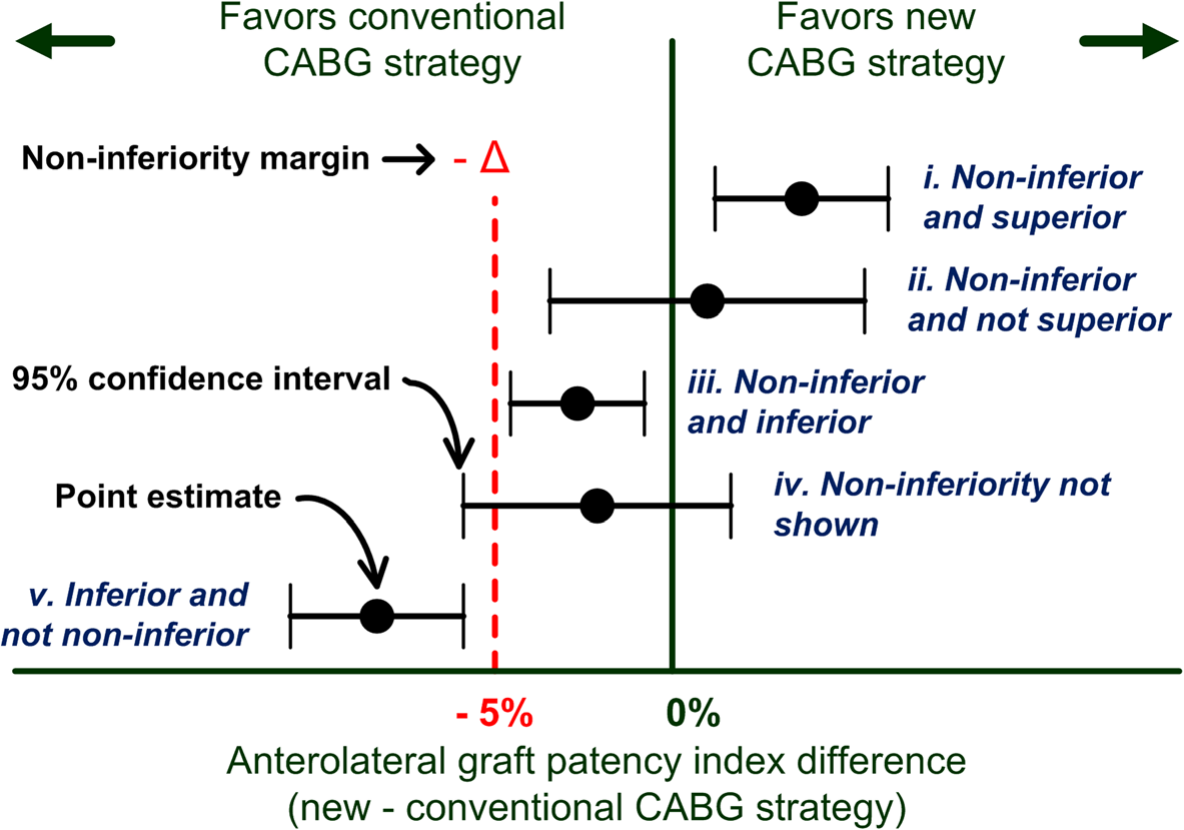
**Possible trial outcomes.** Potential study outcomes are presented with point estimate for the difference in anterolateral graft patency index between the two study groups with a 95% confidence interval for the difference. See text for details. CABG, coronary artery bypass grafting.

According to a non-inferiority study design, five trial outcomes are possible (Figure 4). For non-inferiority to be demonstrated (cases i, ii, and iii), the point estimate and the 95% confidence interval of the difference in anterolateral graft patency index between the new and conventional grafting group have to be greater than the non-inferiority margin of −5%. If non-inferiority is shown, three situations are possible: 1) non-inferiority and superiority, in which the point estimate and the confidence interval completely stand over 0% (case i); 2) non-inferiority and non-superiority, in which the point estimate lies above −5% and the confidence interval includes 0%, but not −5% (case ii); 3) non-inferiority and inferiority, in which the point estimate and the entire confidence interval are between −5% and 0% (case iii). Non-inferiority will not be shown if the confidence interval includes both the −5% non-inferiority margin and the 0% lines (case iv). Finally, inferiority will be established if the point estimate and the confidence interval completely stand below the −5% line (case v).

### Limitations

The trial potential limitations are addressed as follows. First, surgeons, staff of the operating room and of the postoperative yards, personnel collecting the intraoperative data, and patients, will not be blinded to the surgical strategy performed. However, as the two CABG techniques are regularly practiced at our institution and their postoperative managements are similar, we do not foresee any impact of this non-blinding on the results. Second, to limit the operator bias, surgeries will be performed by four surgeons who perform both surgical strategies on a routine basis with an average of more than a hundred CABG surgeries done each year, and the surgical technique will be clearly standardized between surgeons. Moreover, as randomization will be stratified by surgeon, each surgeon will practice as many of both surgical strategies. Third, in order to limit the clinical outcomes assessment bias, each outcome is clearly defined and determined by blinded evaluators. Fourthly, as the primary outcome, anterolateral patency index at one year, is a combined outcome, interpretation bias could arise from the fact that it will be difficult to distinguish from this composite measure if the occluded graft sections (LAD or non LAD segment) differ according to the strategy used. To remedy to this potential bias, patency of each graft taken separately will be assessed as a secondary outcome. Finally, patients with renal failure and/or calcified aorta will be excluded because of contraindication to MSCT and impossibility to practice both type of CABG surgery, respectively. Although they could beneficiate from a LSVB strategy, this trial will not provide data specifically for these populations.

### Trial potential impact

If the trial demonstrates the non-inferiority of the novel grafting strategy, it could have an important impact on the adult cardiac surgery field, given the potential benefits of the LSVB technique. As we are faced with increasingly older patients presenting important comorbidities and poorer coronary targets, this population could benefit from this surgical alternative, allowing in some patient subsets a more optimal operation potentially leading to better overall results.

## Trial status

The recruitment began in July 2012 and is expected to take 3 to 4 years (ending in 2015 to 2016).

## Abbreviations

CABG: Coronary artery bypass grafting; CCS: Canadian cardiovascular society; CPB: Cardiopulmonary bypass; ECG: Electrocardiogram; LAD: Left anterior descending coronary artery; LIMA: Left internal mammary artery; LSVB: Left internal mammary artery-saphenous vein bridge; MI: Myocardial infarction; MSCT: Multislice computed tomography; NYHA: New York heart association; SVG: Saphenous vein graft.

## Competing interests

The authors declare that they have no competing interests.

## Authors’ contributions

LMS conceived and designed the study, including the statistical elements and power calculation. The study protocol and manuscript were written and critically reviewed by AD, NN, CCL, GS, SM, JAT, FD, IP and LMS. All authors read and approved the final manuscript.
